# Differential representation of liver proteins in obese human subjects suggests novel biomarkers and promising targets for drug development in obesity

**DOI:** 10.1080/14756366.2017.1292262

**Published:** 2017-03-08

**Authors:** Simonetta Caira, Antonio Iannelli, Rosaria Sciarrillo, Gianluca Picariello, Giovanni Renzone, Andrea Scaloni, Pietro Addeo

**Affiliations:** a Proteomics and Mass Spectrometry Laboratory, ISPAAM, National Research Council, Naples, Italy;; b Département de Chirurgie Digestive, Centre Hospitalier Universitarie de Nice, Nice, France;; c Dipartimento di Scienze e Tecnologie, Università degli Studi del Sannio, Benevento, Italy;; d Institute of Food Sciences, National Research Council, Avellino, Italy;; e Service de Chirurgie Hépatique, Pancréatique, Biliaire et Transplantation, Pôle des Pathologies Digestives, Hépatiques et de la Transplantation, Hôpital de Hautepierre, Université de Strasbourg, Hôpitaux Universitaires de Strasbourg, Strasbourg, France

**Keywords:** Obesity, human liver proteome, protein biomarkers, oxidative stress, metabolic syndrome

## Abstract

The proteome of liver biopsies from human obese (O) subjects has been compared to those of nonobese (NO) subjects using two-dimensional gel electrophoresis (2-DE). Differentially represented proteins were identified by matrix-assisted laser desorption/ionization time-of-flight (MALDI-TOF) mass spectrometry (MS)-based peptide mass fingerprinting (PMF) and nanoflow-liquid chromatography coupled to electrospray-tandem mass spectrometry (nLC-ESI-MS/MS). Overall, 61 gene products common to all of the liver biopsies were identified within 65 spots, among which 25 ones were differently represented between O and NO subjects. In particular, over-representation of short-chain acyl-CoA dehydrogenase, Δ(3,5)-Δ(2,4)dienoyl-CoA isomerase, acetyl-CoA acetyltransferase, glyoxylate reductase/hydroxypyruvate reductase, fructose-biphosphate aldolase B, peroxiredoxin I, protein DJ-1, catalase, α- and β-hemoglobin subunits, 3-mercaptopyruvate S-transferase, calreticulin, aminoacylase 1, phenazine biosynthesis-like domain-containing protein and a form of fatty acid-binding protein, together with downrepresentation of glutamate dehydrogenase, glutathione S-transferase A1, S-adenosylmethionine synthase 1A and a form of apolipoprotein A-I, was associated with the obesity condition. Some of these metabolic enzymes and antioxidant proteins have already been identified as putative diagnostic markers of liver dysfunction in animal models of steatosis or obesity, suggesting additional investigations on their role in these syndromes. Their differential representation in human liver was suggestive of their consideration as obesity human biomarkers and for the development of novel antiobesity drugs.

## Introduction

Obesity is often prodromal to severe degenerative liver diseases that can also progress to cirrhosis and hepatocellular carcinoma. It has been suggested that obese individuals undergo a progressive oxidative stress, due to increased levels of reactive oxygen and nitrogen species (ROS and RNS). The intricate mechanisms contributing to oxidative stress have been recently reviewed^1^. The net result is that the oxidative stress leads to fat accumulation in nonadipose tissues, with subsequent development of obesity-associated comorbidities. Evidences for a general ROS involvement have been provided without the identification of the initiating events at cellular level. Finally, inflammatory processes modify the plasma levels of alanine transaminase, aspartate transaminase (AST), alkaline phosphatase, γ-glutamyl transferase and bilirubin, which are common plasma markers of the liver obesity status. However, these biomarkers are secreted also by other organs in response to pathological events and, hence, are nonspecific predictors of the liver damage. Thus, novel biomarkers are necessary to improve the accuracy of the diagnosis and to predict the undergoing dysfunction. The discovery of protein signatures may also disclose early-stage disease, supporting the screening of high-risk subjects and grounding the most appropriate treatment.

Clinical proteomics is deeply improving the relationships between proteomes, syndromes and diseases. In this context, quantitative proteomics recently allowed defining tentative protein patterns in the liver associated with specific diseases, including hepatocarcinoma (HCC)^2–6^. Regarding nonalcoholic steatosis (NAS), nonalcoholic fatty liver disease (NAFLD) and obesity, most of the proteomic studies performed so far were realized on hepatic extracts from animal models of the diseases, including *ob*/*ob* mice and hypertensive corpulent rats subjected to a high-fat diet (HFD) or treated with drugs inducing steatosis^7–20^. In contrast, only few preliminary proteomic investigations have been performed on human patients, due to the difficulties in having corresponding liver specimens^21–23^. The present study was aimed at obtaining further insights into the liver protein changes associated with obesity by performing a proteomic comparison of biopsies from O and NO subjects, which was realized with integrated 2-DE, MS and bioinformatics techniques.

## Methods

### Sample of liver biopsies

Wedge liver biopsies were taken at the time of bariatric surgery (laparoscopic Roux-en-Y gastric bypass) or colorectal surgery for the lean control, at the beginning of the procedure. Three out of 10 patients underwent surgery for colorectal disease including diverticular disease (*n*= 2) and colorectal adenoma (*n* =1). The latter had no previous chemotherapy neither liver metastases.

Clinical information and liver samples from subjects with and without obesity were obtained after the approval by the Ethical Committee on Human Research of the participating hospitals and with patient consent. Table 1 reports the baseline clinical characteristics of subjects enrolled in the study. Specimens were taken at the level of the segment III of the liver and were quickly added to anhydrous ethanol, freeze-dried and stored at −80 °C until analysis. A total of 10 biopsies were analyzed in this study. Seven biopsies were from independent patients with obesity with a body mass index (BMI) > 40 and three from independent subjects without obesity (BMI <25) considered as potential candidates for living organ donation. Liver specimens of the subjects without obesity were histologically normal (absence of evident inflammation, fibrosis and pathological pattern).

**Table 1.** Baseline clinical characteristics of the subjects enrolled in this study.

	Group with obesity (*n* = 7)	Lean group (*n* = 3)
Age (median, range)	42 (30–55)	49 (35–51)
Sex (F/M)	4/3	2/1
BMI (median, range)	42 (40–49)	22 (21–25)
Hypertension	3	1
Diabetes	1	0
Sleep apnea	1	0
Dyslipidemia	1	0
Arthrosis	1	0
Bariatric surgery	7	0
Colorectal surgery	0	3

Chemicals and solvents (HPLC-grade or better) were from Sigma-Aldrich (Milan, Italy).

### Protein extraction

Liver biopsies were suspended in liquid nitrogen and ground to a fine powder in ∼10 vol of 700 mM sucrose, 500 mM Tris-HCl, pH 8.0, 50 mM EDTA, 100 mM KCl, 2% v/v β-mercaptoethanol, 1 mM phenylmethylsulfonylfluoride and then vortexed for 15 min at 4 °C. After addition of an equal volume of 500 mM Tris-HCl pH 7.5, the mixtures were vortexed for additional 10 min, centrifuged at 10,000×*g* for 15 min at 4 °C and kept overnight at –20 °C in cold saturated ammonium acetate and methanol. Insoluble proteins were pelleted at 10,000×*g* for 30 min, washed twice with cold methanol and then with cold acetone. Resulting dried pellets were re-suspended in 9 M urea, 4% w/v CHAPS, 0.5% v/v Triton X-100, 20 mM DTT and 1% w/v ampholyte pH 3–10 (BioRad, Hercules, CA), extensively vortexed and finally centrifuged at 10,000×*g* for 10 min, at 20 °C. Protein content was determined from the resulting supernatant using the RC/DC protein assay (Bio-Rad, Hemel Hempstead, UK). Aliquots (100 or 400 μg) of hepatic protein extracts were separated by analytical or preparative 2-DE gels, respectively.

### Two-dimensional electrophoresis (2-DE)

First dimension isoelectric focusing (IEF) was performed on a Protean IEF Cell (Bio-Rad, Hercules, CA) using 18-cm ready IPG strips with a 3–10 linear pH gradient (Bio-Rad). Protein extracts were loaded onto the strips and soaked in the rehydration solution (final volume 315 μL) containing 8 M urea, 2% w/v 3-[(3-cholamidopropyl)dimethylammonio]-1-propanesulfonate, 0.3% w/v dithiotreitol, 2% IPG buffer pH 3–10 and 0.002% w/v Bromophenol Blue for 16 h, at 22 °C. IEF was performed by applying a voltage of 250 V for 1 h, ramping to 1000 V over 5 h and holding at 8000 V until a total of 52 kVh was reached. Then, gel strips were equilibrated in 6 M urea, 30% w/v glycerol, 2% w/v SDS, 50 mM Tris-HCl pH 8.8, 0.01% w/v bromophenol blue and 2% w/v DTT for 20 min. Second dimension SDS-PAGE was carried out in 12% polyacrylamide gels (18 cm ×24 cm ×1 mm) in 25 mM Tris-HCl pH 8.3, 1.92 M glycine and 1% w/v SDS, using a Protean apparatus (Bio-Rad, Hercules, CA), with 70 V (l35 mA) applied for 16 h. Three technical replicate analyses (including sample extraction and 2-DE separation) were carried out for each biological replicate. Gels were stained with Coomassie Brilliant Blue G-250.

### Image acquisition and analysis

Stained gels were scanned at 95.3 μm/pixel resolution using a GS-800 Calibrated Imaging Densitometer (Bio-Rad, Hercules, CA). Resulting 12-bit images were analyzed using the PDQuest software (Bio-Rad, Hercules, CA, v.7.1). Spot detection and matching between gels were performed automatically, followed by manual validation. Only protein spots detected in all the gels were considered for the master gel construction. For quantitative analysis, the spot densities were normalized against the whole-gel densities, and the percentage volume of each spot was averaged for six different gels (3 replicates of 2 biological samples from each individual). Individual spot quantities were thus expressed as parts per million of the total integrated absorbance, after normalization against total image density. Statistical analysis (by Student’s *t*-test) was performed to find out significant protein representation differences between O and NO samples (control). A minimum 2-fold change in normalized spot densities was considered indicative for a differentially represented component. Each pattern was compared to the reference gel of the NO subjects. Constant spots occurring in all gel patterns were used as landmarks to facilitate image matching. Raw quantitative data for each spot were statistically analyzed and intergroup fold differences were calculated. The dynamic range of the protein expression was roughly estimated for 25 spots of each sample using the Image J analysis software (Bio-Rad, Hercules, CA). Quantitative data were obtained by normalizing the volume of each spot with respect to the corresponding one in the control. Statistical comparison of the densitometric data was carried out using the Student’s test for samples, and results were expressed as means ± standard deviation using SPSS 16.0 (SPSS Inc., Chicago, IL). Statistical significance was set at *p*< .05.

### Spot digestion with trypsin

Gel spots were manually excised, reduced, alkylated with iodoacetamide and digested with proteomic-grade trypsin (Promega, Madison, WI)^24^. Protein digests were desalted/concentrated with μZipTip C_18_ devices (Millipore) prior to MALDI-TOF MS and/or nLC-ESI-MS/MS analysis.

### Mass spectrometry

MALDI-TOF-PMF experiments were carried out on a Voyager DE-Pro instrument (PerSeptive Biosystems, Framingham, MA), using α-cyano-4-hydroxycinnamic acid (10 mg/mL in 50%, acetonitrile/0.1% trifluoroacetic acid) as the matrix. For PMF-based identification, spectra were acquired in the positive reflector ion mode. External mass calibration was performed with a dedicated kit of standard peptides (Sigma). Data were elaborated using the DataExplorer 5.1 software (Applied Biosystems, Foster City, CA). 

nLC-ESI-MS/MS analyzes were carried out using an Easy-nanoLC (Proxeon, Odense, Denmark) coupled to a LTQ XL mass spectrometer (Thermo Finnigan, San Jose, CA) equipped with a Proxeon nanospray source^25^. Peptide mixtures were separated on a Proxeon Easy C_18_ column (100 × 0.075 mm, 3 μm) using a gradient of 0.1% v/v formic acid in acetonitrile and 0.1% formic acid in water. Acetonitrile ramped from 5% to 35% over 15 min and from 35% to 95% over 2 min, at a flow rate of 300 nL/min. Acquisition was controlled by a data-dependent product ion scanning procedure over the three most abundant ions, enabling dynamic exclusion (repeat count 2 and exclusion duration 1 min). The mass isolation window and collision energy were *m/z* 3 and 35%, respectively.

### Protein identification

MASCOT software package version 2.2.06 (Matrix Science, UK) was used to identify protein spots from an updated nonredundant sequence database (NCBI nr 01/2013). MALDI-TOF-PMF data were searched using a mass tolerance value of 40–80 ppm, trypsin as proteolytic enzyme, up to two allowed missed cleavages, Cys carbamidomethylation as fixed modification, Met-oxidation and N-terminal Gln-conversion to pyroglutamate as variable modifications. nLC-ESI-MS/MS data were employed to interrogate NCBI database, using a mass tolerance value of 2 Da for precursor ion and 0.8 Da for MS/MS fragments and proteolytic enzyme, missed cleavage and fixed/variable modification information reported earlier. Searches were taxonomically restricted to human proteins. MALDI-TOF-PMF candidates with a cumulative MASCOT score >82 and/or nLC-ESI-MS/MS candidates with more than two assigned peptides with an individual MASCOT score >25, both corresponding to *p* <0.05 for a significant identification, were further evaluated by the comparison with their calculated mass and *pI* values, using the experimental values obtained from 2-DE. Protein identification was validated by measuring corresponding false positive discovery rate values (lower than 1%).

### Functional classification of the differentially expressed proteins

Differentially represented proteins were examined according to the Protein ANalysis THrough Evolutionary Relationship (PANTHER) application (http://www.pantherdb.org) to standardize the representation of gene product attributes across species and databases. Sequence information was used to classify a gene to an ontology group according to the Gene Ontology (GO) terms (http://www.geneontology.org). Differentially represented proteins were clustered to GO according to their subcellular localization, primary biological process and molecular function.

## Results and discussion

### Liver proteome comparison between obese and nonobese subjects

The human liver proteome is strikingly complex; more than 6800 gene products have been described so far, which still represent a portion of the predicted 11,000 expressed genes^26^^,^^27^. Due to the technological approach we used in the present study, our analysis was limited to proteins having a high-medium representation level in the liver, which were quantitatively evaluated as candidate obesity biomarkers. Worth mentioning is the fact that the specimens were from O and NO organs that did not show any pathological feature when histologically inspected; the latter came from lean subjects considered as potential candidates for living liver donation.

**Figure 1.**
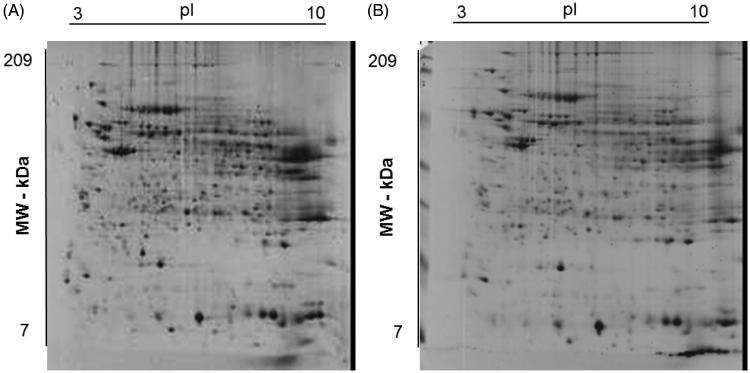
Representative Coomassie G250 Blue-stained 2-D-gel pattern of a human liver biopsy homogenate from subject with (A) and without (B) obesity.

**Figure 2.**
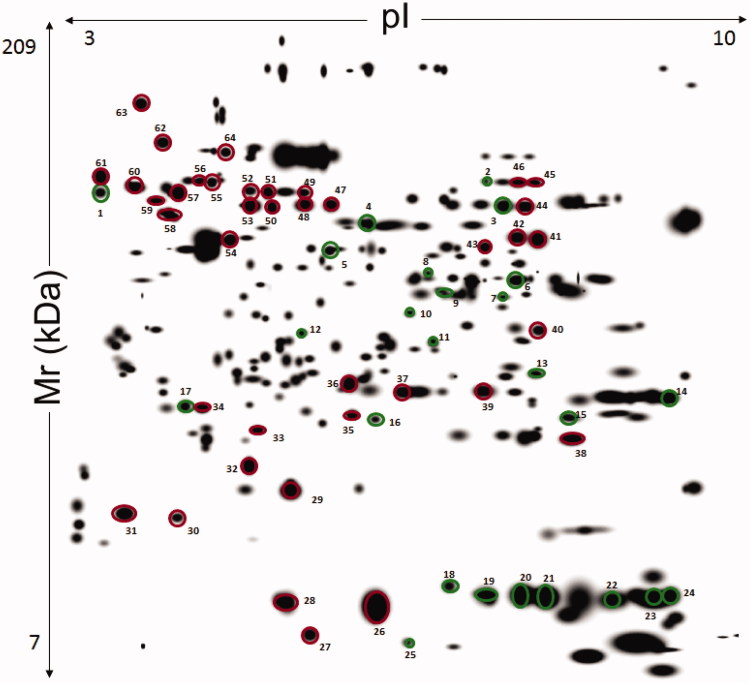
Master gel from 2-DE experiments on liver extracts of human subjects with and without obesity. The resulting 12-bit images were automatically analyzed using PDQuest software based on the spots identified across the gels of liver extract from seven patients with obesity and three without obesity. First dimension, IEF pH 3 to 10 NL (left to right); second dimension, SDS-containing 12% T polyacrylamide gel electrophoresis. Spot numbering corresponds to that reported in Table 2. The spots from 1 to 25 indicate a different quantitative representation with respect to control. Spots from 26 to 64 that were common to all compared samples and did not show significant differences.

Some differences were observed amongst the O (panel A) and NO (panel B) subjects in explorative 2-DE analytical comparisons (Figure 1). This finding was confirmed by preparative experiments performed by analyzing higher protein amounts, which better highlighted some differentially represented proteins between O and NO subjects. In particular, although O subjects showed proteomic patterns affected by a certain individual variability (see below), a common quantitative trend was observed for specific proteins with respect to control. Protein spot features across all the gels were imported into a composite master gel (Figure 2). In this figure, green-circled features (25 in number) correspond to spots that significantly changed their representation between O and NO groups, while red-circled ones did not show significant variations among O/NO specimens. In the whole, 21 spots occurred as over-represented in the O group, whereas four ones were downrepresented.

### Identification of liver proteins

Proteins present in the green and red-circled spots were identified by MALDI-TOF-PMF and/or nLC-ESI-MS/MS analysis and were associated with 61 sequence entries (Table 2). Identification details are reported in Supplementary Table S1. Among the differentially represented spots in the O group, 23 ones contained a unique protein species, whereas two (spots 6 and 9) showed two components that comigrated within the gel as result of their similar mass and *pI* values. The volume data corresponding to the 25 differentially represented spots provided a rough estimation of the dynamic range of protein expression among the different O individuals (Table 3). Coherent data (at least 5 over 7 individuals) were observed for about 76% of the variable spots.

**Table 2.** Mass spectrometry-based identification of proteins identified in the liver proteomes of obese (O) and nonobese (NO) subjects.

Spot	Proteins	SWISS PROT entry	NCBI entry	Spot	Proteins	SWISS PROT entry	NCBI entry
**1**	Calreticulin	CALR_HUMAN	4757900	**43**	Isocitrate dehydrogenase [NADP] cytoplasmic	IDHC_HUMAN	28178825
**2**	Catalase	CATA_HUMAN	4557014		4-hydroxyphenylpyruvate dioxygenase	HPPD_HUMAN	4504477
**3**	Glutamate dehydrogenase 1, mitochondrial	DHE3_HUMAN	4885281	**44**	Glutamate dehydrogenase 1, mitochondrial	DHE3_HUMAN	4885281
**4**	S-adenosylmethioninesynthetase isoform type-1	METK1_HUMAN	417297	**45**	Catalase	CATA_HUMAN	4557014
**5**	Aminoacylase-1	ACY1_HUMAN	461466		Delta-1-pyrroline-5-carboxylate dehydrogenase, mitochondrial	AL4A1_HUMAN	25777734
**6**	Aspartate aminotransferase, cytoplasmic	AATC_HUMAN	5902703	**46**	Catalase	CATA_HUMAN	4557014
	Acetyl-CoA acetyltransferase, mitochondrial	THIL_HUMAN	135755		Delta-1-pyrroline-5-carboxylate dehydrogenase, mitochondrial	AL4A1_HUMAN	25777734
**7**	Fructose-bisphosphate aldolase B	ALDOB_HUMAN	40354205	**47**	Aldehyde dehydrogenase, mitochondrial	ALDH2_HUMAN	25777732
**8**	Short-chain-specific acyl-CoA dehydrogenase, mitochondrial	ACADS_HUMAN	4557233	**48**	Aldehyde dehydrogenase, mitochondrial	ALDH2_HUMAN	25777732
**9**	Aldo-keto reductase family 1 member C4	AK1C4_HUMAN	1705823	**49**	Protein disulfide-isomerase A3	PDIA3_HUMAN	21361657
	Glyoxylate reductase/hydroxypyruvate reductase	GRHPR_HUMAN	47116943	**50**	Keratin, type II cytoskeletal 8	K2C8_HUMAN	4504919
**10**	3-mercaptopyruvate sulfurtransferase	THTM_HUMAN	61835204	**51**	Formimidoyltransferase-cyclodeaminase	FTCD_HUMAN	11140815
**11**	Phenazine biosynthesis-like domain-containing protein	PBLD_HUMAN	62177133	**52**	Formimidoyltransferase-cyclodeaminase	FTCD_HUMAN	11140815
**12**	Delta(3,5)-Delta(2,4)-dienoyl-CoA isomerase, mitochondrial	ECH1_HUMAN	82654933	**53**	Keratin, type II cytoskeletal 8	K2C8_HUMAN	4504919
**13**	Carbonic anhydrase 2	CAH2_HUMAN	4557395	**54**	Keratin, type I cytoskeletal 18	K1C18_HUMAN	4557888
**14**	Glutathione S-transferase A1	GSTA1_HUMAN	22091454	**55**	60 kDa heat shock protein, mitochondrial	CH60_HUMAN	31542947
**15**	Peroxiredoxin-1	PRDX1_HUMAN	548453	**56**	60 kDa heat shock protein, mitochondrial	CH60_HUMAN	31542947
**16**	Protein DJ-1	PARK7_HUMAN	31543380	**57**	Vimentin	VIME_HUMAN	62414289
**17**	Apolipoprotein A-I	APOA1_HUMAN	4557321		Tubulin alpha-1B chain	TBA1B_HUMAN	193787715
**18**	Hemoglobin subunit beta	HBB_HUMAN	4504349	**58**	ATP synthase subunit beta, mitochondrial	ATPB_HUMAN	32189394
**19**	Hemoglobin subunit beta	HBB_HUMAN	4504349	**59**	Tubulin beta chain	TBB5_HUMAN	18088719
**20**	Hemoglobin subunit beta	HBB_HUMAN	4504349	**60**	Protein disulfide-isomerase	PDIA1_HUMAN	2507460
**21**	Hemoglobin subunit beta	HBB_HUMAN	4504349		Alpha-1-antitrypsin	A1AT_HUMAN	1703025
**22**	Fatty acid-binding protein, liver	FABPL_HUMAN	4557577	**61**	Calreticulin	CALR_HUMAN	4757900
**23**	Hemoglobin subunit alpha	HBA_HUMAN	4504345	**62**	78 kDa glucose-regulated protein	GRP78_HUMAN	16507237
**24**	Hemoglobin subunit alpha	HBA_HUMAN	4504345	**63**	Endoplasmin	ENPL_HUMAN	4507677
**25**	Pterin-4-alpha-carbinolamine dehydratase	PHS_HUMAN	4557831	**64**	Annexin A6	ANXA6_HUMAN	113962
**26**	Fatty acid-binding protein, liver	FABPL_HUMAN	4557577		Heat shock cognate 71 kDa protein	HSP7C_HUMAN	123648
**27**	Acyl-CoA-binding protein	ACBP_HUMAN	118276		Serum albumin	ALBU_HUMAN	4502027
	Hemoglobin subunit beta	HBB_HUMAN	4504349		V-type proton ATPase catalytic subunit A	VATA_HUMAN	22096378
	Cytochrome c oxidase subunit 6B1	CX6B1_HUMAN	117115				
	GTP cyclohydrolase 1 feedback regulatory protein	GFRP_HUMAN	2506906				
	Fatty acid-binding protein, liver	FABPL_HUMAN	4557577				
**28**	Fatty acid-binding protein, liver	FABPL_HUMAN	4557577				
**29**	Superoxide dismutase [Cu-Zn]	SODC_HUMAN	4507149				
**30**	Eukaryotic translation initiation factor 5A-1	IF5A1_HUMAN	54037409				
	Glia maturation factor beta	GMFB_HUMAN	46577593				
**31**	Cytochrome b5	CYB5_HUMAN	41281768				
**32**	Ferritin light chain	FRIL_HUMAN	20149498				
**33**	Peroxiredoxin-2	PRDX2_HUMAN	32189392				
	Ferritin light chain	FRIL_HUMAN	20149498				
**34**	Apolipoprotein A-I	APOA1_HUMAN	4557321				
**35**	Ferritin light chain	FRIL_HUMAN	20149498				
	Thioredoxin-dependent peroxide reductase, mitochondrial	PRDX3_HUMAN	2507171				
**36**	Enoyl-CoA hydratase, mitochondrial	ECHM_HUMAN	1922287				
	Heat shock protein beta-1	HSPB1_HUMAN	4504517				
**37**	Peroxiredoxin-6	PRDX6_HUMAN	4758638				
**38**	Phosphatidylethanolamine-binding protein 1	PEBP1_HUMAN	4505621				
**39**	Glutathione S-transferase Mu 1	GSTM1_HUMAN	23065544				
**40**	Thiosulfate sulfurtransferase	THTR_HUMAN	17402865				
**41**	Betaine–homocysteineS-methyltransferase 1	BHMT1_HUMAN	157266337				
**42**	Betaine–homocysteineS-methyltransferase 1	BHMT1_HUMAN	157266337				

The first 25 spots reports proteins identified as differentially represented in the O subjects.

**Table 3.** Proteins identified as over- and down-represented in the liver of O subjects, compared to control (NO subjects).

Spot	1	2	3	4	5	6	7	8	9	10	11	12	13	14	15	16	17	18	19	20	21	22	23	24	25
Protein	Calreticulin	Catalase	Glutamate dehydrogenase 1	S-adenosylmethionine synthetase isoform type 1	Aminoacylase-1	Acetyl-CoA acetyltransferase, mitochondrial; aspartate aminotransferase, cytoplasmic	Fructose- bisphosphate aldolase B	Short-chain specific acyl-CoA dehydrogenase, mitochondrial	Aldo-keto reductase family 1 member C4; Glyoxylate reductase/ hydroxypyruvate	3-Mercaptopyruvate sulfurtransferase	Phenazine biosynthesis-like domain- containing protein	Δ(3,5)- Δ(2,4)-dienoyl-CoA isomerase, mitochondrial	Carbonic anhydrase 2	Glutathione S-transferase A1	Peroxiredoxin-1	Protein DJ-1	Apolipoprotein A-I	Hemoglobin subunit beta	Hemoglobin subunit beta	Hemoglobin subunit beta	Hemoglobin subunit beta	Fatty acid-binding protein	Hemoglobin subunit alpha	Hemoglobin subunit alpha	Pterin-4-alpha-carbinolamine dehydratase
Sample																									
1	–	++	–	–	+++	+	++	++	++		+++	+++	+	–	+	+	–	–	–	–	–	–	–	++	++
2	+	++	–	–	+	+	+	+	–	+++	++	++	++	–	–	++	–	+	++++	+++	++	++++	+	+++++	+
3	++++	+++	–	–	+	+	+++	–	++		–	–	–	–	+++	+++	–	+	+++	++	++	+	+	+	++
4	–	+	–	–	++	–	–	++	+++	+	+	++	+	–	+	–	–	–	++++	+	++	+	++	++	–
5	+	–	–	–	–	+++++	–	–	++	+	+	–	++	–	++	+	–	+	–	++	+++		–	+	++
6	+	+++	–	–	++	–	+	+	–	+	–	+	–	–	+	++	–	++	–	++	++	++	+++++	++	++
7	+++++	–	–	+	+	+	++++	+++	++	+	++	+	+	+++	+	+	–	+	–	–	+	+	+	++	–
+	>0 < 5		–	>0 < −5																					
++	>5 < 10		–	>−5 < −10																					
+++	>10 < 15		–	>−10 < −15																					
++++	>15 < 20		–	>−15 < −20																					
+++++	>20		–	>−20																					

The protein value was calculated by subtracting the average volume value of the NO subjects (assumed as reference data) to that of each measured spot. The protein in the liver biopsies was judged to be positive or negative according to the higher or lower value than 0 score in comparison to the reference samples. Volume measurements (expressed as percentage) were indicated with + or – signs, depending on the degree of protein expression.

**Figure 3.**
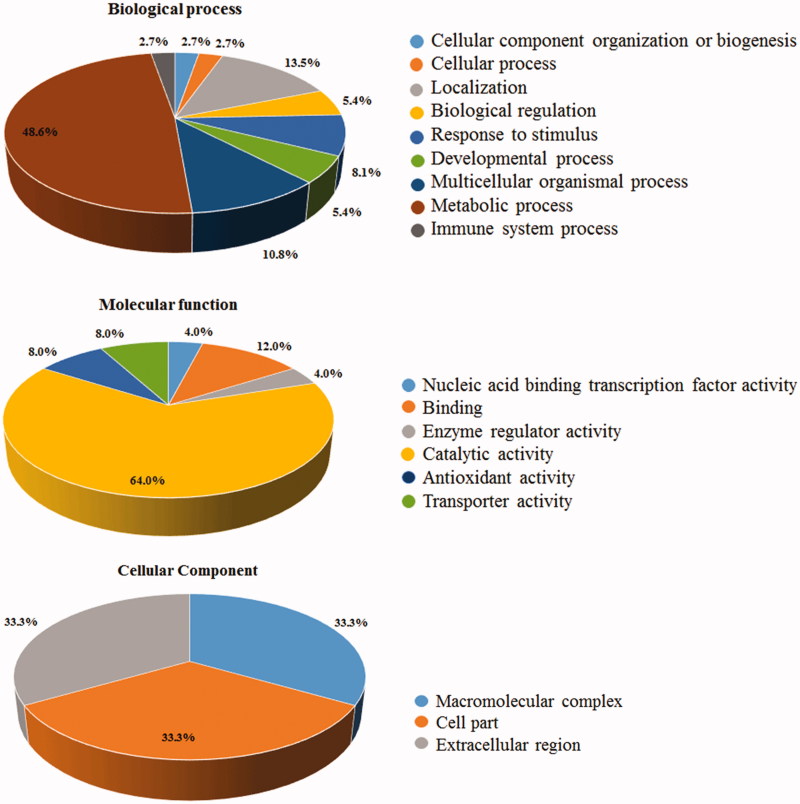
Gene Ontology (GO) annotation of identified proteins. The graphs show the percentages of corresponding GO terms to the 25 differentially represented components. The identified proteins were clustered according to three different criteria: cellular component, biological process, and molecular function.

Differentially represented proteins were clustered according to GO terms for the corresponding molecular function, biological process and cellular component (Figure 3). More than 50% of the entries corresponded to protein effectors involved in metabolic processes, among which 60% corresponded to fatty acid-/ phospholipid-binding proteins. Notably, nearly 10% of the differentially represented proteins were inflammation-related factors.

### Differentially represented proteins in obese subjects

**Figure 4.**
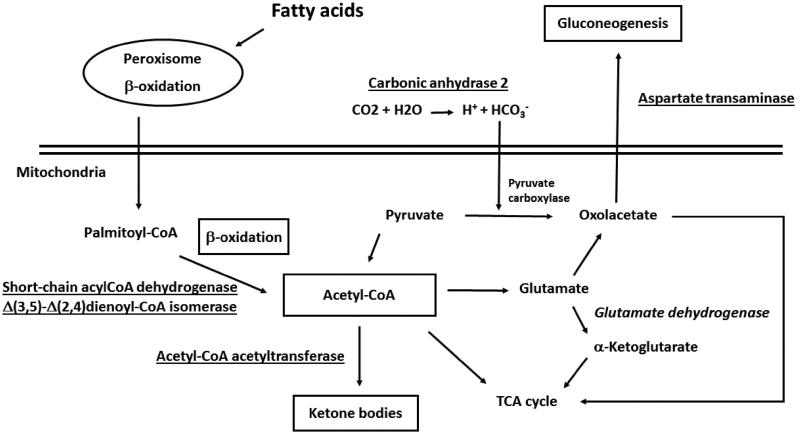
Enzymes involved in fatty acid β-oxidation, ketogenesis, pyruvate metabolism and gluconeogenesis and acetyl-CoA generation/consumption deregulated process in human subjects with obesity. Over-represented proteins and promoted biological processes were underlined; down-represented proteins and reduced biological processes were in italics.

Recent observations already pointed out that no a unique molecule may be used as a marker candidate for obesity in human plasma, but rather a subset of components^28^. These findings found a counterpart in results from various proteomic studies realized on liver tissues of animal models of steatosis, NAFLD and obesity^7–20^. In this context, although exhibiting a variety of functions in the liver, most of the proteins here identified as differentially represented in O subjects have already been characterized as coherently deregulated in the liver of *ob*/*ob* mice and corpulent rats subjected to a HFD or treated with steatosis-inducing drugs. For example, increased levels of mitochondrial short-chain acyl-CoA dehydrogenase (spot 8) and Δ(3,5)-Δ(2,4)dienoyl-CoA isomerase (spot 12) (Table 3) have already been ascertained in above-mentioned rodent models^9^^,^^10^^,^^13^^,^^14^^,^^18^. Augmented protein amounts were also verified in the case of acetyl-CoA acetyltransferase (spot 6)^10^^,^^13^, glyoxylate reductase/hydroxypyruvate reductase (spot 9)^13^ and AST (spot 6)^10^^,^^14^^,^^17^^,^^19^ (Table 3). Thus, over-representation of enzymes involved in fatty acid β-oxidation, ketogenesis, pyruvate metabolism and gluconeogenesis was suggestive for a deregulated process of acetyl-CoA generation/consumption in human O subjects (Figure 4), where the accumulation of this metabolite, as result of the augmented oxidative degradation of fatty acids and a higher metabolic activity of liver mitochondria, is redirected toward other metabolic pathways^10^^,^^11–13^^,^^17^^,^^20^. The over-representation of fructose-biphosphate aldolase B (spot 7), which assists production of trioses phosphate for glycogen replenishment, and the down-representation of glutamate dehydrogenase (spot 3), which links together acetyl-CoA, α-ketoglutarate and TCA cycle, were also in line with this scenario. Coherent quantitative levels of these enzymes have already been observed in murine models^9^^,^^14^^,^^17^^,^^18^ and obese humans^21^^,^^29^ (Table 3). Recently, short-chain acyl-CoA dehydrogenase, AST, fructose-biphosphate aldolase B and glutamate dehydrogenase have been identified as components of a multiprotein complex, containing other mitochondrial and cytosolic proteins, which integrates the different pathways mentioned above and possibly acts as an interpaths master controller^30^. Also the over-representation of carbonic anhydrase 2 (spot 13) in O subjects (Table 3) should be easily rationalized according to the picture reported in Figure 4, since this enzyme contributes to provide a significant portion of the bicarbonate for pyruvate carboxylase-assisted conversion of pyruvate into oxaloacetate in adipose metabolism^31^. Increased levels of this protein have been associated with the process of adipocyte differentiation/maturation^32^^,^^33^. In this context, this and other carbonic anhydrases have been identified as targets for the treatment and prophylaxis of obesity^34^.

Regarding fatty acids and cholesterol transporters, which have already been described as differentially represented in the liver of the above-mentioned rodent models to face the increased concentration of these metabolites and to facilitate the corresponding metabolism/excretion^5^^,^^14^^,^^17^^,^^21^, no quantitative changes were observed for spots associated with apolipoprotein A-I (apoA-I) and fatty acid-binding protein (FABP-1) (spots 26, 27, 28 and 34). Unique exceptions were specific protein isoforms (spots 17 and 22) (Table 3), which have already been associated with an acid apoA-I form bearing oxidized Met and Trp residues observed in a murine model of HCC^35^, and with a basic carbonylated FABP-1 form containing HNE- or glutathione-modified amino acids detected in a murine model of alcoholic liver disease^36^^,^^37^, respectively. These modified species were reported to have a reduced transporter activity^36^^,^^38^. Their differential representation in the liver of O subjects either matches with previous biochemical reports on rodent models^14^^,^^17^^,^^33^^,^^39^ and humans^23^^,^^39^^,^^40^ and was suggestive of an increased ROS activity therein^1^^,^^6^^,^^21^^,^^41^^,^^42^. Accordingly, they may be considered as tentative pathological hallmarks that might help to decipher the traits of the abnormal lipid metabolism in obesity.

The observation of a ROS increase in the liver of O subjects^1^^,^^6^^,^^37^^,^^42^ found a parallel in the measured over-representation of proteins involved in the response to oxidative insult, namely peroxiredoxin (PRX) I (spot 15), protein DJ-1 (spot 16) and catalase (CAT) (spot 2) (Table 3). For the latter protein, augmented levels were detected only for the most acid isoform, which corresponds to its phosphorylated, activated form^43^. These and other antioxidant proteins have already been reported as over-represented in above-mentioned rodent models^6^^,^^10^^,^^15^^,^^17^^,^^18^^,^^42^. Their upregulation was ascribed to an attempt of the organ to contrast the oxidative burst generated from overloaded, fatty liver mitochondria^1^^,^^6^^,^^42^. PRXs are abundant peroxidases that, through their thioredoxin peroxidase activity, maintain low levels of endogenous H_2_O_2_^44^^,^^45^; DJ-1 is an oxidative stress sensor protecting cells against oxidative insult^46^^,^^47^; CAT is a peroxisomal antioxidant enzyme deputed to the inactivation of H_2_O_2_ with quantitative levels modulated from cell redox state^45^. Since it has been reported that the oxidative burst can be considered either as first or second hit triggering hepatosteatotis development^1^^,^^6^^,^^42^, it would be interesting to evaluate the quantitative evolution of these antioxidant proteins in subjects with incipient obesity.

The over-representation of α- and β-hemoglobin subunits (spots 18–21 and 23–24) in O subjects (Table 3) was also associated with an antioxidant function. These proteins have already been identified as upregulated in the hepatocytes of NAS and NAFLD subjects^48^. Their over-representation was not associated with erythropoiesis, but as related to the increased oxidative stress affecting the liver of the patients^1^^,^^6^^,^^42^. In particular, hemoglobin was demonstrated to reduce this redox insult as result of its antioxidative peroxidase activity^48^^,^^49^. Both hemoglobin chains have already been proposed as plasma biomarkers for staging the liver damage^50^.

Glutathione S-transferase (GST)-A1 (spot 14) and GST-Mu1 (spot 39) were also characterized as down-represented or constant in O specimens, respectively (Table 3). Reduced levels of these enzymes have already been observed in above-mentioned rodent models^9^^,^^14^^,^^51^ and in patients suffering these systemic metabolic alterations^21^^,^^22^^,^^52^. GSTs are liver-detoxifying enzymes that catalyze the conjugation of toxic xenobiotics and lipid peroxidation products with glutathione (GSH)^43^. Since augmented levels of lipid peroxides and reduced levels of GSH have already been associated with a HFD^21^^,^^29^^,^^53^^,^^54^, a reduced GSTs activity would not effectively counteract increased levels of lipid peroxides in obese subjects, thus determining a significant protein oxidative modification, mitochondrial dysfunction and tissue damage^42^^,^^55^.

Other enzymes involved in the metabolism of S-containing derivatives here observed as differentially represented in O subjects were S-adenosylmethionine synthase 1A (MAT1A, down-represented, spot 4) and 3-mercaptopyruvate S-transferase (3-MST, over-represented, spot 10) (Table 3). Their quantitative levels well paralleled with that observed in the rodent models of metabolic dysfunction mentioned above^20^^,^^21^^,^^42^^,^^55^. In particular, MAT1A is an essential enzyme for liver metabolism that catalyzes formation of S-adenosylmethionine (adoMet), an intracellular methylating effector and essential precursor for GSH biosynthesis. This protein helps maintaining normal liver functions, since MAT1A chronic hepatic deficiency results in the spontaneous development of nonalcoholic steatohepatitis, oxidative stress and HCC^15^^,^^42^. Our findings further support the important role of this protein in obesity-associated liver syndromes. On the other hand, 3-MST mediates the production of H_2_S from L-Cys in liver mitochondia, generating a gas that maintain organelle electron flow and supports cellular energetics^56^^,^^57^. Our results provide a further evidence for the direct relation between the enhanced H_2_S-producing capacity of obese livers and the over-representation of 3-MST, which in this context should act as an antioxidant/anti-inflammatory molecule^56^^,^^57^.

Finally, a positive correlation of our quantitative results with data from human and/or murine models of liver (metabolic) diseases was also observed for calreticulin (spot 1)^58^^,^^59^, aminoacylase 1 (spot 13)^60^ and phenazine biosynthesis-like domain-containing protein (spot 11)^61^ (Table 3). An over-representation of the endoplasmic reticulum stress-marker calreticulin has already been associated with the inflammation affecting patients having systemic metabolic alterations and HFD^62^. Aminoacylase 1 is Zn-dependent enzyme that hydrolyzes Ac-amino acids (including the antioxidant Ac-Cys) into acetate and amino acids^63^; its increased representation was observed in the liver of a mouse model suffering inflammatory conditions^60^ and in a condition of Ac-Cys depletion^62^. It was demonstrated that aminoacylase forms a regulatory/functional network with transketolase^63^ and interacts directly with sphingosine kinase 1^64^; both protein couples have been proposed to have a role in the onset of obesity.

## Conclusions

In this study, various proteins have been identified as differentially represented in the liver of O subjects, compared to control. Despite the low number of biopsies analyzed as result of the difficulties in collecting it, the nature of deregulated proteins matched that of differentially represented components ascertained in animal models of associated metabolic syndromes^7–20^ or in preliminary investigations on human obese patients^21–23^. We considered this observation as an independent validation of our results and, based on the reduced availability of corresponding tissues, it prompted us to avoid performing confirmative western blotting assays. In this context, the limited number of deregulated enzymes here detected, compared to that generally ascertained in animal models^7–20^, may be the consequence of human to mouse differences, reflect a softer metabolic disfunction of the human subjects and/or depend on technical limitations associated with the liver amounts or to the gel-based approach we used.

Most of the components here identified as deregulated were associated with protein families already ascertained as highly affected in obesity-associated syndromes in rodent models^7–20^, namely metabolic enzymes and proteins involved in the response to an oxidative stress, confirming their role also in the human syndrome. For the first class, protein identity suggested that quantitative changes occurred at mitochondrial and cytosolic levels, and affected various metabolic pathways (fatty acid β-oxidation, ketogenesis, pyruvate metabolism and gluconeogenesis) involved in acetyl-CoA generation/consumption. These findings support previous studies reporting that obesity in humans is associated with a marked metabolic disfunction in the liver, which may be dependent on a cellular redox imbalance at mitochondrial level^1^^,^^6^^,^^42^. Various hypothesis have been proposed regarding the possibility that the oxidative insult may be prodromal or additional for organ dysfunction. The observed over-representation of various antioxidant enzymes was in line with this scenario and was confirmative of the oxidative stress hepatocytes from obese subjects experienced and the reduced GSH levels present therein^21^^,^^29^^,^^53^^,^^54^. The nature of some FBP-1 and APOA-I isoforms, as detected here, suggested that some proteins become the target of oxidative modifications in obese subjects. In this context, a number of studies have already reported increased glutathionylated and carbonylated protein levels in animal models of NAFLD and NAS^6^^,^^42^^,^^45^^,^^55^^,^^65–68^. Future redox proteomics investigations combined with functional assays will clarify the dynamics of these modifications and their effect on the active proteome of the liver in obese patients.

Specific differentially represented proteins (FBP-1, APOA-I and hemoglobin chains) transport lipids/nutrients within organs, cells or cell compartments. Some were reported to influence lipid homeostasis and inflammation, playing a role in the onset of obesity^40^^,^^69^^,^^70^. Their quantitative changes in human liver as result of the obesity income (Table 3) well paralleled with that already reported in plasma^40^^,^^48^^,^^50^^,^^70^. Thus, our data further support their use as clinically relevant biomarkers for obesity-like metabolic dysfunctions. Future studies are needed to validate the other deregulated proteins here identified and to link their levels to additional plasma biomarkers.

In conclusion, this study identified some metabolic enzymes and antioxidant proteins that have already been identified as putative diagnostic markers of liver dysfunction in animal models of steatosis or obesity. Their differential representation in human liver was suggestive of their consideration as potential biomarkers also for humans. Their quantitative representation suggests novel studies aimed at investigating the effect of their possible inhibition on obesity dysfunction; in this context, their identification can be considered as prodrome to the development of novel antiobesity drugs. In this context, some synthetic molecules have already been proposed as selective inhibitors of some of these enzymes and thus as antiobesity drugs. In particular, topiramate and zonisamide have already been reported as CA II inhibitors based on their capability to bind Zn^2+^ in the enzyme active site^71^. Similarly, 2-aminobicyclo-(2,2,1)-heptane-2-carboxylic acid (BCH) is a nonmetabolized analog of leucine that acts as a strong allosteric activator of GDH^72^. It stimulates the reductive amination through GDH activation thus reducing both *de novo* lipogenesis and gluconeogenesis. BCH has also been demonstrated to reduce liver collagen and plasma levels of alanine transaminase and aspartate transaminase. Finally, avasimibe has been reported to inhibit acetyl-CoA acyltransferase, thus reducing plasma total cholesterol and VLDL concentrations with an unknown mechanism.

## Supplementary Material

IENZ_1292262_SI.pdf

## References

[1] Savini I, Catani MV, Evangelista D, et al Obesity-associated oxidative stress: strategies finalized to improve redox state. Int J Mol Sci 2013;14:10497–538. 23698776 10.3390/ijms140510497 PMC3676851

[2] Matos JM, Witzmann FA, Cummings OW, Schmidt CM. A pilot study of proteomic profiles of human hepatocellular carcinoma in the United States. J Surg Res 2009;155:237–43. 19535095 10.1016/j.jss.2008.06.008 PMC2859432

[3] Codarin E, Renzone G, Poz A, et al Differential proteomic analysis of subfractioned human hepatocellular carcinoma tissues. J Proteome Res 2009;8:2273–84. 19290626 10.1021/pr8009275

[4] Cesaratto L, Vascotto C, D'Ambrosio C, et al Overoxidation of peroxiredoxins as an immediate and sensitive marker of oxidative stress in HepG2 cells and its application to the redox effects induced by ischemia/reperfusion in human liver. Free Radic Res 2005;39:255–68. 15788230 10.1080/10715760400029603

[5] Kimhofer T, Fye H, Taylor-Robinson S, et al Proteomic and metabonomic biomarkers for hepatocellular carcinoma: a comprehensive review. Br J Cancer 2015;112:1141–56. 25826224 10.1038/bjc.2015.38 PMC4385954

[6] Scaloni A, Codarin E, Di Maso V, et al Modern strategies to identify new molecular targets for the treatment of liver diseases: The promising role of proteomics and redox proteomics investigations. Clin Appl 2009;3:242–62. 10.1002/prca.200800169 26238622

[7] Takahashi Y, Soejima Y, Fukusato T. Animal models of nonalcoholic fatty liver disease/nonalcoholic steatohepatitis. World J Gastroenterol 2012;18:2300–8. 22654421 10.3748/wjg.v18.i19.2300 PMC3353364

[8] Meneses-Lorente G, Watt A, Salim K, et al Identification of early proteomic markers for hepatic steatosis. Chem Res Toxicol 2006;19:986–98. 16918237 10.1021/tx060007f

[9] Zhang X, Yang J, Guo Y, et al Functional proteomic analysis of nonalcoholic fatty liver disease in rat models: enoyl-coenzyme a hydratase down-regulation exacerbates hepatic steatosis. Hepatology 2010;51:1190–9. 20162621 10.1002/hep.23486

[10] Douette P, Navet R, Gerkens P, et al Steatosis-induced proteomic changes in liver mitochondria evidenced by two-dimensional differential in-gel electrophoresis. J Proteome Res 2005;4:2024–31. 16335947 10.1021/pr050187z

[11] Meierhofer D, Weidner C, Sauer S. Integrative analysis of transcriptomics, proteomics, and metabolomics data of white adipose and liver tissue of high-fat diet and rosiglitazone-treated insulin-resistant mice identified pathway alterations and molecular hubs. J Proteome Res 2014;13:5592–602. 25287014 10.1021/pr5005828

[12] Guo Y, Darshi M, Ma Y, et al Quantitative proteomic and functional analysis of liver mitochondria from high fat diet (HFD) diabetic mice. Mol Cell Proteomics 2013;12:3744–58. 24030101 10.1074/mcp.M113.027441 PMC3861721

[13] Edvardsson U, von Löwenhielm HB, Panfilov O, et al Hepatic protein expression of lean mice and obese diabetic mice treated with peroxisome proliferator-activated receptor activators. Proteomics 2003;3:468–78. 12687614 10.1002/pmic.200390061

[14] Santamaria E, Avila MA, Latasa MU, et al Functional proteomics of nonalcoholic steatohepatitis: mitochondrial proteins as targets of S-adenosylmethionine. Proc Natl Acad Sci USA 2003;100:3065–70. 12631701 10.1073/pnas.0536625100 PMC152247

[15] Wang X, Choi JW, Oh TS, et al Comparative hepatic proteome analysis between lean and obese rats fed a high-fat diet reveals the existence of gender differences. Proteomics 2012;12:284–99. 22140079 10.1002/pmic.201100271

[16] Hölper S, Nolte H, Bober E, et al Dissection of metabolic pathways in the Db/Db mouse model by integrative proteome and acetylome analysis. Mol Biosyst 2015;11:908–22. 25592279 10.1039/c4mb00490f

[17] Sabidó E, Wu Y, Bautista L, et al Targeted proteomics reveals strain-specific changes in the mouse insulin and central metabolic pathways after a sustained high-fat diet. Mol Syst Biol 2013;9:681. 23860498 10.1038/msb.2013.36 PMC3734509

[18] Meneses-Lorente G, Guest PC, Lawrence J, et al A proteomic investigation of drug-induced steatosis in rat liver. Chem Res Toxicol 2004;17:605–12. 15144217 10.1021/tx034203n

[19] Chang J, Oikawa S, Ichihara G, et al Altered gene and protein expression in liver of the obese spontaneously hypertensive/NDmcr-cp rat. Nutr Metab 2012;9:87. 10.1186/1743-7075-9-87 PMC3565951 22998770

[20] Eccleston HB, Andringa KK, Betancourt AM, et al Chronic exposure to a high-fat diet induces hepatic steatosis, impairs nitric oxide bioavailability, and modifies the mitochondrial proteome in mice. Antioxid Redox Signal 2011;15:447–59. 20919931 10.1089/ars.2010.3395 PMC3118652

[21] Valle A, Catalán V, Rodríguez A, et al Identification of liver proteins altered by type 2 diabetes mellitus in obese subjects. Liver Int 2012;32:951–61. 22340678 10.1111/j.1478-3231.2012.02765.x

[22] Younossi ZM, Baranova A, Ziegler K, et al A genomic and proteomic study of the spectrum of nonalcoholic fatty liver disease. Hepatology 2005;42:665–74. 16116632 10.1002/hep.20838

[23] Charlton M, Viker K, Krishnan A, et al Differential expression of lumican and fatty acid binding protein-1: new insights into the histologic spectrum of nonalcoholic fatty liver disease. Hepatology 2009;49:1375–84. 19330863 10.1002/hep.22927 PMC2674237

[24] Shevchenko A, Tomas H, Havlis J, et al In-gel digestion for mass spectrometric characterization of proteins and proteomes. Nat Protoc 2006;1:2856–60. 17406544 10.1038/nprot.2006.468

[25] Scippa GS, Rocco M, Ialicicco M, et al The proteome of lentil (*Lens culinaris* Medik.) seeds: discriminating between landraces. Electrophoresis 2010;31:497–506. 20119961 10.1002/elps.200900459

[26] Chinese Human Liver Proteome Profiling Consortium: First insight into the human liver proteome from PROTEOME(SKY)-LIVER(Hu) 1.0, a publicly available database. J Proteome Res 2010;9:79–94. 19653699 10.1021/pr900532r

[27] Sun A, Jiang Y, Wang X, et al Liverbase: a comprehensive view of human liver biology. J Proteome Res 2010;9:50–8. 19670857 10.1021/pr900191p

[28] Pearce SG, Thosani NC, Pan J-J. Noninvasive biomarkers for the diagnosis of steatohepatitis and advanced fibrosis in NAFLD. Biomarker Res 2013;1:1–11. 10.1186/2050-7771-1-7 PMC4177607 24252302

[29] Lee SB, Cho HI, Jin YW, et al Wild ginseng cambial meristematic cells ameliorate hepatic steatosis and mitochondrial dysfunction in high-fat diet-fed mice. J Pharm Pharmacol 2016;68:119–27. 26806698 10.1111/jphp.12487

[30] Narayan SB, Master SR, Sireci AN, et al Short-chain 3-hydroxyacyl-coenzyme A dehydrogenase associates with a protein super-complex integrating multiple metabolic pathways. PLoS One 2012;7:e35048. 22496890 10.1371/journal.pone.0035048 PMC3322157

[31] Hazen SA, Waheed A, Sly WS, et al Differentiation-dependent expression of CA V and the role of carbonic anhydrase isozymes in pyruvate carboxylation in adipocytes. FASEB J 1996;10:481–90. 8647347 10.1096/fasebj.10.4.8647347

[32] Lynch CJ, Hazen SA, Horetsky RL, et al Differentiation-dependent expression of carbonic anhydrase II and III in 3T3 adipocytes. Am J Physiol 1993;265:C234–243. 8338133 10.1152/ajpcell.1993.265.1.C234

[33] Kheterpal I, Ku G, Coleman L, et al Proteome of human subcutaneous adipose tissue stromal vascular fraction cells versus mature adipocytes based on DIGE. J Proteome Res 2011;10:1519–27. 21261302 10.1021/pr100887r PMC3070065

[34] De Simone G, Supuran CT. Antiobesity carbonic anhydrase inhibitors. Curr Top Med Chem 2007;7:879–84. 17504132 10.2174/156802607780636762

[35] Fernández-Irigoyen J, Santamaría E, Sesma L, et al Oxidation of specific methionine and tryptophan residues of apolipoprotein A-I in hepatocarcinogenesis. Proteomics 2005;5:4964–72. 16252306 10.1002/pmic.200500070

[36] Dörmann P, Börchers T, Korf U, et al Amino acid exchange and covalent modification by cysteine and glutathione explain isoforms of fatty acid-binding protein occurring in bovine liver. J Biol Chem 1993;268:16286–92. 8344916

[37] Smathers RL, Galligan JJ, Stewart BJ, Petersen DR. Overview of lipid peroxidation products and hepatic protein modification in alcoholic liver disease. Chem Biol Interact 2011;192:107–12. 21354120 10.1016/j.cbi.2011.02.021 PMC3109208

[38] Smathers RL, Fritz KS, Galligan JJ, et al Characterization of 4-HNE modified L-FABP reveals alterations in structural and functional dynamics. PLoS One 2012;7:e38459. 22701647 10.1371/journal.pone.0038459 PMC3368874

[39] Yang F, Yin Y, Wang F, et al An altered pattern of liver apolipoprotein A-I isoforms is implicated in male chronic hepatitis B progression. J Proteome Res 2010;9:134–43. 19788185 10.1021/pr900593r

[40] Atshaves BP, Martin GG, Hostetler HA, et al Liver fatty acid-binding protein and obesity. J Nutr Biochem 2010;21:1015–32. 20537520 10.1016/j.jnutbio.2010.01.005 PMC2939181

[41] Cesaratto L, Vascotto C, Calligaris S, Tell G. The importance of redox state in liver damage. Ann Hepatol 2004;3:86–92. 15505592

[42] Mantena SK, King AL, Andringa KK, et al Mitochondrial dysfunction and oxidative stress in the pathogenesis of alcohol- and obesity-induced fatty liver diseases. Free Radic Biol Med 2008;44:1259–72. 18242193 10.1016/j.freeradbiomed.2007.12.029 PMC2323912

[43] Rhee SG, Yang KS, Kang SW, et al Controlled elimination of intracellular H_2_O_2_: regulation of peroxiredoxin, catalase, and glutathione peroxidase via post-translational modification. Antioxid Redox Signal 2005;7:619–26. 15890005 10.1089/ars.2005.7.619

[44] Latimer HR, Veal EA. Peroxiredoxins in regulation of MAPK signalling pathways; sensors and barriers to signal transduction. Mol Cells 2016;39:40–5. 26813660 10.14348/molcells.2016.2327 PMC4749872

[45] Bachi A, Dalle-Donne I, Scaloni A. Redox proteomics: chemical principles, methodological approaches and biological/biomedical promises. Chem Rev 2013;113:596–698. 23181411 10.1021/cr300073p

[46] Knobbe CB, Revett TJ, Bai Y, et al Choice of biological source material supersedes oxidative stress in its influence on DJ-1 in vivo interactions with Hsp90. J Proteome Res 2011;10:4388–404. 21819105 10.1021/pr200225c PMC5006933

[47] Taira T, Saito Y, Niki T, et al DJ-1 has a role in antioxidative stress to prevent cell death. EMBO Rep 2004;5:213–18. 14749723 10.1038/sj.embor.7400074 PMC1298985

[48] Liu W, Baker SS, Baker RD, et al Upregulation of haemoglobin expression by oxidative stress in hepatocytes and its implication in non-alcoholic steatohepatitis. PLoS One 2011;6:e24363. 21931690 10.1371/journal.pone.0024363 PMC3171444

[49] Xu L, Xu CF, Yu CH, et al Haemoglobin and non-alcoholic fatty liver disease: further evidence from a population-based study. Gut 2009;58:1706–7. 19923352 10.1136/gut.2009.186668

[50] Trak-Smayra V, Dargere D, Noun R, et al Serum proteomic profiling of obese patients: correlation with liver pathology and evolution after bariatric surgery. Gut 2009;58:825–32. 18403495 10.1136/gut.2007.140087

[51] Noeman SA, Hamooda HE, Baalash AA. Biochemical study of oxidative stress markers in the liver, kidney and heart of high fat diet induced obesity in rats. Diabetol Metab Syndr 2011;3:17. 21812977 10.1186/1758-5996-3-17 PMC3174870

[52] Kurzawski M, Dziedziejko V, Urasińska E, et al Nuclear factor erythroid 2-like 2 (Nrf2) expression in end-stage liver disease. Environ Toxicol Pharmacol 2012;34:87–95. 22459801 10.1016/j.etap.2012.03.001

[53] Tsuchiya H, Ebata Y, Sakabe T. High-fat, high-fructose diet induces hepatic iron overload via a hepcidin-independent mechanism prior to the onset of liver steatosis and insulin resistance in mice. Metabolism 2013;62:62–9. 22854109 10.1016/j.metabol.2012.06.008

[54] Böhm T, Berger H, Nejabat M, et al Food-derived peroxidized fatty acids may trigger hepatic inflammation: a novel hypothesis to explain steatohepatitis. J Hepatol 2013;59:563–70. 23665282 10.1016/j.jhep.2013.04.025

[55] Curtis JM, Grimsrud PA, Wright WS, et al Downregulation of adipose glutathione S-transferase A4 leads to increased protein carbonylation, oxidative stress, and mitochondrial dysfunction. Diabetes 2010;59:1132–42. 20150287 10.2337/db09-1105 PMC2857893

[56] Rashid S, Alexander S, Roberts R. Enhanced synthesis of hydrogen sulfide in liver and kidney from Zucker diabetic Fatty rats (ZDF) compared to Zucker Lean (ZL) rats. Nitric Oxide 2013;31:S54.

[57] Módis K, Coletta C, Erdélyi K, et al Intramitochondrial hydrogen sulfide production by 3-mercaptopyruvate sulfurtransferase maintains mitochondrial electron flow and supports cellular bioenergetics. FASEB J 2013;27:601–11. 23104984 10.1096/fj.12-216507

[58] Zhang W, Ambati S, Della-Fera MA, et al Leptin modulated changes in adipose tissue protein expression in ob/ob mice. Obesity (Silver Spring) 2011;19:255–61. 20725060 10.1038/oby.2010.166

[59] Boden G, Duan X, Homko C, et al Increase in endoplasmic reticulum stress-related proteins and genes in adipose tissue of obese, insulin-resistant individuals. Diabetes 2008;57:2438–44. 18567819 10.2337/db08-0604 PMC2518495

[60] Labbus K, Henning M, Borkham-Kamphorst E, et al Proteomic profiling in lipocalin 2 deficient mice under normal and inflammatory conditions. J Proteomics 2013;78:188–96. 23219901 10.1016/j.jprot.2012.11.021

[61] Li A, Yan Q, Zhao X, et al Decreased expression of PBLD correlates with poor prognosis and functions as a tumor suppressor in human hepatocellular carcinoma. Oncotarget 2016;7:524–37. 26594798 10.18632/oncotarget.6358 PMC4808015

[62] Calzadilla P, Gómez-Serrano M, García-Santos E, et al N-Acetylcysteine affects obesity-related protein expression in 3T3-L1 adipocytes. Redox Rep 2013;18:210–18. 24112955 10.1179/1351000213Y.0000000066 PMC6837643

[63] Pérez-Pérez R, García-Santos E, Ortega-Delgado FJ, et al Attenuated metabolism is a hallmark of obesity as revealed by comparative proteomic analysis of human omental adipose tissue. J Proteomics 2012;75:783–95. 21989264 10.1016/j.jprot.2011.09.016

[64] Maceyka M, Nava VE, Milstien S, Spiegel S. Aminoacylase 1 is a sphingosine kinase 1-interacting protein. FEBS Lett 2004;568:30–4. 15196915 10.1016/j.febslet.2004.04.093

[65] Hu X, Duan Z, Hu H, et al Proteomic profile of carbonylated proteins in rat liver: exercise attenuated oxidative stress may be involved in fatty liver improvement. Proteomics 2013; 13:1755–64. 23505022 10.1002/pmic.201200522

[66] Deng Z, Yan S, Hu H, et al Proteomic profile of carbonylated proteins in rat liver: discovering possible mechanisms for tetracycline-induced steatosis. Proteomics 2015;15:148–59. 25332112 10.1002/pmic.201400115

[67] Curtis JM, Hahn WS, Stone MD, et al Protein carbonylation and adipocyte mitochondrial function. J Biol Chem 2012;287:32967–80. 22822087 10.1074/jbc.M112.400663 PMC3463318

[68] Piemonte F, Petrini S, Gaeta LM, et al Protein glutathionylation increases in the liver of patients with non-alcoholic fatty liver disease. J Gastroenterol Hepatol 2008;23:e457–64. 17683488 10.1111/j.1440-1746.2007.05070.x

[69] Shi J, Zhang Y, Gu W, et al Serum liver fatty acid binding protein levels correlate positively with obesity and insulin resistance in Chinese young adults. PLoS One 2012;7:e48777. 23144966 10.1371/journal.pone.0048777 PMC3492433

[70] Bril F, Sninsky JJ, Baca AM, et al Hepatic Steatosis and Insulin Resistance, But Not Steatohepatitis, Promote Atherogenic Dyslipidemia in NAFLD. J Clin Endocrinol Metab 2016;101:644–52. 26672634 10.1210/jc.2015-3111

[71] Supuran CT, Di Fiore A, De Simone G. Oncologic, endocrine and metabolic carbonic anhydrase inhibitors as emerging drugs for the treatment of obesity. Expert Opin Emerging Drugs 2008;13:383–92. 10.1517/14728214.13.2.383 18537527

[72] Han SJ, Choi SE, Yi SA, et al Glutamate dehydrogenase activator BCH stimulating reductive amination prevents high fat/high fructose diet-induced steatohepatitis and hyperglycemia in C57BL/6J mice. Sci Rep 2016;5:37468. 27874078 10.1038/srep37468 PMC5118703

