# Supplementary material for: Differential representation of liver proteins in obese human subjects suggests novel biomarkers and promising targets for drug development in obesity
Source: J Enzyme Inhib Med Chem. 2017 Mar 8;32(1):672–82. doi: 10.1080/14756366.2017.1292262 (PMC6009959; doi:10.1080/14756366.2017.1292262)
Supplement: IENZ_1292262_SI.pdf [file IENZ_A_1292262_SM1993.pdf]

Supporting Information

Table S1. Identification details of the proteins present in subjects with and without obesity. PMF, MALDI-TOF MS peptide mass fingerprinting; TMS, nLC-ESI-MS/MS. The first twenty-five spots reports proteins identified as differentially represented in the subjects with obesity.

Spot	Protein name	SWISS PROT accession	NCBI accession	pI/Mw theoretical	MS method	n. pept.	Sequence coverage (%)	Mascot Score
1	Calreticulin	CALR_HUMAN	4757900	4,29/48	TMS	13	36	567
2	Catalase	CATA_HUMAN	4557014	6,90/60	TMS	15	32	712
3	Glutamate dehydrogenase 1, mitochondrial	DHE3_HUMAN	4885281	7,66/62	TMS	18	35	925
4	S-adenosylmethionine synthetase isoform type-1	METK1_HUMAN	417297	5,86/44	TMS	10	29	496
5	Aminoacylase-1	ACY1_HUMAN	461466	5,77/46	TMS	14	40	677
6	Aspartate aminotransferase, cytoplasmic	AATC_HUMAN	5902703	6,52/46	TMS	13	37	663
	Acetyl-CoA acetyltransferase, mitochondrial	THIL_HUMAN	135755	8,98/45	TMS	10	29	486
7	Fructose-bisphosphate aldolase B	ALDOB_HUMAN	40354205	8,00/40	PMF	16	47	11
8	Short-chain specific acyl-CoA dehydrogenase, mitochondrial	ACADS_HUMAN	4557233	8,13/45	PMF	16	56	182
9	Aldo-keto reductase family 1 member C4	AK1C4_HUMAN	1705823	6,71/37	TMS	17	55	731
	Glyoxylate reductase/hydroxypyruvate reductase	GRHPR_HUMAN	47116943	7,01/36	TMS	12	40	597
10	3-Mercaptopyruvate sulfurtransferase	THTM_HUMAN	61835204	6,13/33	PMF	20	68	293
11	Phenazine biosynthesis-like domain-containing protein	PBLD_HUMAN	62177133	6,06/32	PMF	18	57	208
12	Δ(3,5)-Δ(2,4)-dienoyl-CoA isomerase, mitochondrial	ECH1_HUMAN	82654933	8,16/36	TMS	11	31	557
13	Carbonic anhydrase 2	CAH2_HUMAN	4557395	6,87/29	PMF	14	58	174
14	Glutathione S-transferase A1	GSTA1_HUMAN	22091454	8,91/26	PMF	23	66	258
15	Peroxiredoxin-1	PRDX1_HUMAN	548453	8,27/22	TMS	17	64	585
16	Protein DJ-1	PARK7_HUMAN	31543380	6,33/20	PMF	12	66	166
17	Apolipoprotein A-I	APOA1_HUMAN	4557321	5,56/31	PMF	16	51	172
18	Hemoglobin subunit beta	HBB_HUMAN	4504349	6,75/16	PMF	11	89	183
19	Hemoglobin subunit beta	HBB_HUMAN	4504349	6,75/16	PMF	16	95	276
20	Hemoglobin subunit beta	HBB_HUMAN	4504349	6,75/16	PMF	16	95	280
21	Hemoglobin subunit beta	HBB_HUMAN	4504349	6,75/16	PMF	16	95	266
22	Fatty acid-binding protein, liver	FABPL_HUMAN	4557577	6,60/14	TMS	5	42	243

23	Hemoglobin subunit alpha	HBA_HUMAN	4504345	8,72/15	PMF	10	83	143
24	Hemoglobin subunit alpha	HBA_HUMAN	4504345	8,72/15	PMF	10	83	143
25	Pterin-4-alpha-carbinolamine dehydratase	PHS_HUMAN	4557831	6,28/12	PMF	10	73	158
26	Fatty acid-binding protein, liver	FABPL_HUMAN	4557577	6,60/14	PMF	16	82	229
27	Acyl-CoA-binding protein	ACBP_HUMAN	118276	6,12/10	TMS	8	68	338
	Hemoglobin subunit beta	HBB_HUMAN	4504349	6,75/16	TMS	4	38	308
	Cytochrome c oxidase subunit 6B1	CX6B1_HUMAN	117115	6,54/10	TMS	4	56	252
	GTP cyclohydrolase 1 feedback regulatory protein	GFRP_HUMAN	2506906	6,08/10	TMS	3	60	187
	Fatty acid-binding protein, liver	FABPL_HUMAN	4557577	6,60/14	TMS	3	25	182
28	Fatty acid-binding protein, liver	FABPL_HUMAN	4557577	6,60/14	PMF	13	74	190
29	Superoxide dismutase [Cu-Zn]	SODC_HUMAN	4507149	5,70/16	PMF	10	86	175
30	Eukaryotic translation initiation factor 5A-1	IF5A1_HUMAN	54037409	5,08/17	TMS	8	45	540
	Glia maturation factor beta	GMFB_HUMAN	46577593	5,19/17	TMS	3	30	180
31	Cytochrome b5	CYB5_HUMAN	41281768	4,88/15	PMF	8	60	144
32	Ferritin light chain	FRIL_HUMAN	20149498	5,51/20	PMF	15	63	204
33	Peroxiredoxin-2	PRDX2_HUMAN	32189392	5,66/22	PMF	15	62	216
	Ferritin light chain	FRIL_HUMAN	20149498	5,51/20	PMF	9	56	101
34	Apolipoprotein A-I	APOA1_HUMAN	4557321	5,56/31	PMF	22	59	273
35	Ferritin light chain	FRIL_HUMAN	20149498	5,51/20	TMS	10	35	390
	Thioredoxin-dependent peroxide reductase, mitochondrial	PRDX3_HUMAN	2507171	7,67/28	TMS	7	33	303
36	Enoyl-CoA hydratase, mitochondrial	ECHM_HUMAN	1922287	8,34/32	PMF	15	59	164
	Heat shock protein beta-1	HSPB1_HUMAN	4504517	5,98/23	PMF	10	42	114
37	Peroxiredoxin-6	PRDX6_HUMAN	4758638	6,00/25	PMF	18	58	238
38	Phosphatidylethanolamine-binding protein 1	PEBP1_HUMAN	4505621	7,01/21	PMF	19	88	257
39	Glutathione S-transferase Mu 1	GSTM1_HUMAN	23065544	6,24/26	PMF	23	77	322
40	Thiosulfate sulfurtransferase	THTR_HUMAN	17402865	6,77/34	PMF	23	71	317
41	Betaine--homocysteine S-methyltransferase 1	BHMT1_HUMAN	157266337	6,58/45	PMF	14	43	160
42	Betaine--homocysteine S-methyltransferase 1	BHMT1_HUMAN	157266337	6,58/45	PMF	14	41	147
43	Isocitrate dehydrogenase [NADP] cytoplasmic	IDHC_HUMAN	28178825	6,53/47	PMF	21	50	239
	4-hydroxyphenylpyruvate dioxygenase	HPPD_HUMAN	4504477	6,52/45	PMF	12	34	143
44	Glutamate dehydrogenase 1, mitochondrial	DHE3_HUMAN	4885281	7,66/62	PMF	36	55	320
45	Catalase	CATA_HUMAN	4557014	6,90/60	PMF	31	51	277
	Δ-1-pyrroline-5-carboxylate dehydrogenase, mitochondrial	AL4A1_HUMAN	25777734	8,25/62	PMF	17	34	117

46	Catalase	CATA_HUMAN	4557014	6,90/60	PMF	31	51	277
	Δ-1-pyrroline-5-carboxylate dehydrogenase, mitochondrial	AL4A1_HUMAN	25777734	8,25/62	PMF	17	34	117
47	Aldehyde dehydrogenase, mitochondrial	ALDH2_HUMAN	25777732	6,63/57	PMF	22	48	210
48	Aldehyde dehydrogenase, mitochondrial	ALDH2_HUMAN	25777732	6,63/57	PMF	24	50	238
49	Protein disulfide-isomerase A3	PDIA3_HUMAN	21361657	5,98/57	PMF	24	43	261
50	Keratin, type II cytoskeletal 8	K2C8_HUMAN	4504919	5,52/54	PMF	36	63	355
51	Formimidoyltransferase-cyclodeaminase	FTCD_HUMAN	11140815	5,58/60	PMF	37	70	436
52	Formimidoyltransferase-cyclodeaminase	FTCD_HUMAN	11140815	5,58/60	PMF	34	67	421
53	Keratin, type II cytoskeletal 8	K2C8_HUMAN	4504919	5,52/54	PMF	43	67	371
54	Keratin, type I cytoskeletal 18	K1C18_HUMAN	4557888	5,34/48	PMF	36	73	339
55	60 kDa heat shock protein, mitochondrial	CH60_HUMAN	31542947	5,70/61	PMF	30	50	316
56	60 kDa heat shock protein, mitochondrial	CH60_HUMAN	31542947	5,70/61	PMF	27	42	294
57	Vimentin	VIME_HUMAN	62414289	5,06/54	PMF	38	83	352
	Tubulin alpha-1B chain	TBA1B_HUMAN	193787715	4,94/51	PMF	24	56	187
58	ATP synthase subunit beta, mitochondrial	ATPB_HUMAN	32189394	5,26/56	PMF	31	65	267
59	Tubulin beta chain	TBB5_HUMAN	18088719	4,78/50	PMF	29	55	311
60	Protein disulfide-isomerase	PDIA1_HUMAN	2507460	4,76/57	TMS	49	77	2096
	Alpha-1-antitrypsin	A1AT_HUMAN	1703025	5,37/47	TMS	15	36	602
61	Calreticulin	CALR_HUMAN	4757900	4,29/48	PMF	19	48	258
62	78 kDa glucose-regulated protein	GRP78_HUMAN	16507237	5,07/72	PMF	25	37	270
63	Endoplasmin	ENPL_HUMAN	4507677	4,76/93	PMF	42	47	357
64	Annexin A6	ANXA6_HUMAN	113962	5,42/76	TMS	43	58	2176
	Heat shock cognate 71 kDa protein	HSP7C_HUMAN	123648	5,37/71	TMS	39	54	1663
	Serum albumin	ALBU_HUMAN	4502027	5,92/71	TMS	27	43	1233
	V-type proton ATPase catalytic subunit A	VATA_HUMAN	22096378	5,35/69	TMS	15	26	698
